# Sexuality in Later Life and Mental Health Among Older Black Gay Men

**DOI:** 10.1093/geroni/igaa057.1005

**Published:** 2020-12-16

**Authors:** Darlingtina Esiaka, Alice Cheng, Candidus Nwakasi

**Affiliations:** 1 Union College, Union College, New York, United States; 2 Union College, Schenectady, New York, United States; 3 University of Southern Indiana, Evansville, Indiana, United States

## Abstract

Self-acknowledgement and integration of racial and sexual identities are significant to one’s overall sense of identity because of their implications for mental health and wellbeing. These issues are important as one ages because older people experience a wide range of factors that add layers to their ability to (re)integrate subsets of their identity into their overall self-identity such as age and age-related disabilities. This study examined the intersection of race and sexual identities on overall health status in older Black gay men, a demographic group that has historically received less attention. Data from the Social Justice Sexuality (SJS) survey of LGBTQ+ people of color which occurred over a 12-month period in the United States were analyzed. Participants (N=160), 50 years and over, responded to questions about their sexuality, social identity, family dynamics, community connection and engagement, and mental and physical health. Results show an association of mental wellbeing with racial and sexual identities. Further, results show that a strong sense of connection to other sexual minorities is positively associated with mental health in older Black gay men. We discuss the implication of findings for mental health interventions targeting this gendered population.

